# Complete mitochondrial genome of the Alpine Metacarpal-tubercled Toad *Leptobrachella alpina* (Amphibia, Anura, Megophryidae)

**DOI:** 10.1080/23802359.2021.1990149

**Published:** 2021-10-20

**Authors:** Guocheng Shu, Min Yu, Zhongping He, Feng Xie, Xixi Liang

**Affiliations:** a Faculty of Agriculture, Forest and Food Engineering, Yibin University, Yibin, China; b Chengdu Institute of Biology, Chinese Academy of Sciences, Chengdu, China; c Institute of Qinghai-Tibetan Plateau, Southwest Minzu University, Chengdu, China; d University of Chinese Academy of Sciences, Beijing, China; e Institute of Zoology, Chinese Academy of Sciences, Beijing, China

**Keywords:** Megophryidae, *Leptobrachella alpina*, mitochondrial genome, phylogenetic analysis

## Abstract

The complete mitochondrial genome of the *Leptobrachella alpina* Fei, Ye, and Li 1990, was assembled for the first time. The mitogenome of this species was 17,763 bp in length, containing 13 protein-coding genes, two ribosomal RNA genes (12S rRNA and 16S rRNA), 22 transfer RNA genes (tRNA), and a non-coding control region (D-loop). The base content of the mitogenome was that A, T, G, and C occupied 28.5%, 30.8%, 15.1%, and 25.6%, respectively. The phylogenetic analysis was conducted based on 17 complete mitogenome sequences of the family Megophryidae by the Bayesian inference approach. The phylogenetic tree suggested that *Leptobrachium* and *Oreolalax* clustered into a clade and formed a sister group with *Leptobrachella.* This work is critical for the further genetic research and conservation of this species.

*Leptobrachella alpina* is a member of the genus *Leptobrachella* of the family Megophryidae (Fei et al. 2012). It is endemic to China and distributed in Jingdong, Yunnan and Cengwanglaoshan, Tianlin, Guangxi. *L. alpina* lives in a lush vegetation mountainous area at an altitude of 1150–2400 m. The wild population of the species is very small, and its threatened level is Endangered in the Biodiversity Red List of China (Fei et al. 2012).

The specimen of *L. alpina* was collected from Jingdong County, Yunnan Province, China (23.9949 N, 101.5395E). The voucher specimen was deposited in the Herpetological Museum of Chengdu Institute of Biology (Voucher no. YN20130305001, Contact person: Feng Xie, xifeng@cib.ac.cn). Genomic DNA was extracted from muscle tissue using DNeasy Blood & Tissue Kit (TIANGEN). Standard PCR and LA-PCR methods were used to amplify the mitochondrial genome of this species. The complete mitochondrial genome of *L.alpina* was submitted to GenBank with the accession number of MW487804.

The complete mitogenome of *L.alpina* had a total length of 17,763 bp and contains 37 genes, including 13 protein-coding genes, two ribosomal RNA (12S rRNA and 16S rRNA) genes, and 22 transfer RNA (tRNA) genes, and a non-coding control region (D-loop). The base composition of A, G, C, and T in the mitogenome was 28.5%, 15.1%, 25.6%, and 30.8%, respectively. The GC content of the whole mitogenome was 40.7%. *L.alpina* shared roughly similar mtDNA gene arrangement with the most of amphibian species (Xianget al. 2013a; Xiang et al. 2013b; Liu et al. 2015; Xu et al. 2016; Liang et al. 2016a). However, some gene rearrangements have been found in this species. It is similar to species of the genus *Oreolalax* (Liang et al. 2016b), with a tRNA-Met duplication, but they are no longer arranged in a tandem array. The tRNA-Val gene translocated from upstream of 16S rRNA gene to downstream of tRNA-Met1 gene, and the tRNA-Pro gene translocated from downstream of tRNA-Thr to upstream of tRNA-Met2 gene, in short, two tRNA-Met genes were separated by tRNA-Val and tRNA-Pro gene. Besides, the tRNA-Trp gene has lost.

Total 26 genes were encoded on the heavy strand (H-strand), including 12 protein-coding genes and 14 tRNA genes. ND6 gene and 8 tRNA genes were encoded on L-strand. The 12S rRNA and 16S rRNA were located between tRNA-Phe and tRNA-Leu (UUR) genes, with length 934 bp and 1587 bp, respectively. Among protein-coding genes, the shortest was the ATP8 gene with only 168 bp, and the longest was the ND5 gene with 1815 bp.

We constructed the phylogenetic (Figure 1) relationships with 17 complete sequences of the family Megophryidae by Bayesian inference (BI). For BI analyses, three runs and four Markov chains were executed in MrBayes 3.2.2 (Ronquist et al. 2012) using the model (GTR + I + G) and starting from a random tree. Each run was conducted with a total of 5 × 10^6^ generations and sampled every 1000 generations. The first 25% generations were discarded as burn-in, and the remaining samples were used to create a 50% majority rule consensus tree and estimate Bayesian posterior probabilities (BPP). The topology was almost consistent with Liang et al. (2016a) and Liang et al. (2016b) obtained. Namely, all megophryidae species were split into three well-supported major clades. *Leptobrachium* and *Oreolalax* clustered into a clade and formed a sister group with *Leptobrachella*. Finally, the big clade formed a sister group with *Megophrys*. The genetic distance was calculated by MEGA7 (Kumar et al. 2016) with P-distance model. The genetic distance between *L. alpina* and *Megophrys sp* is the largest at 30.40%, and the genetic distance between *L. alpine* and *O. schmidti* is the smallest at 26.75%.

**Figure 1.**
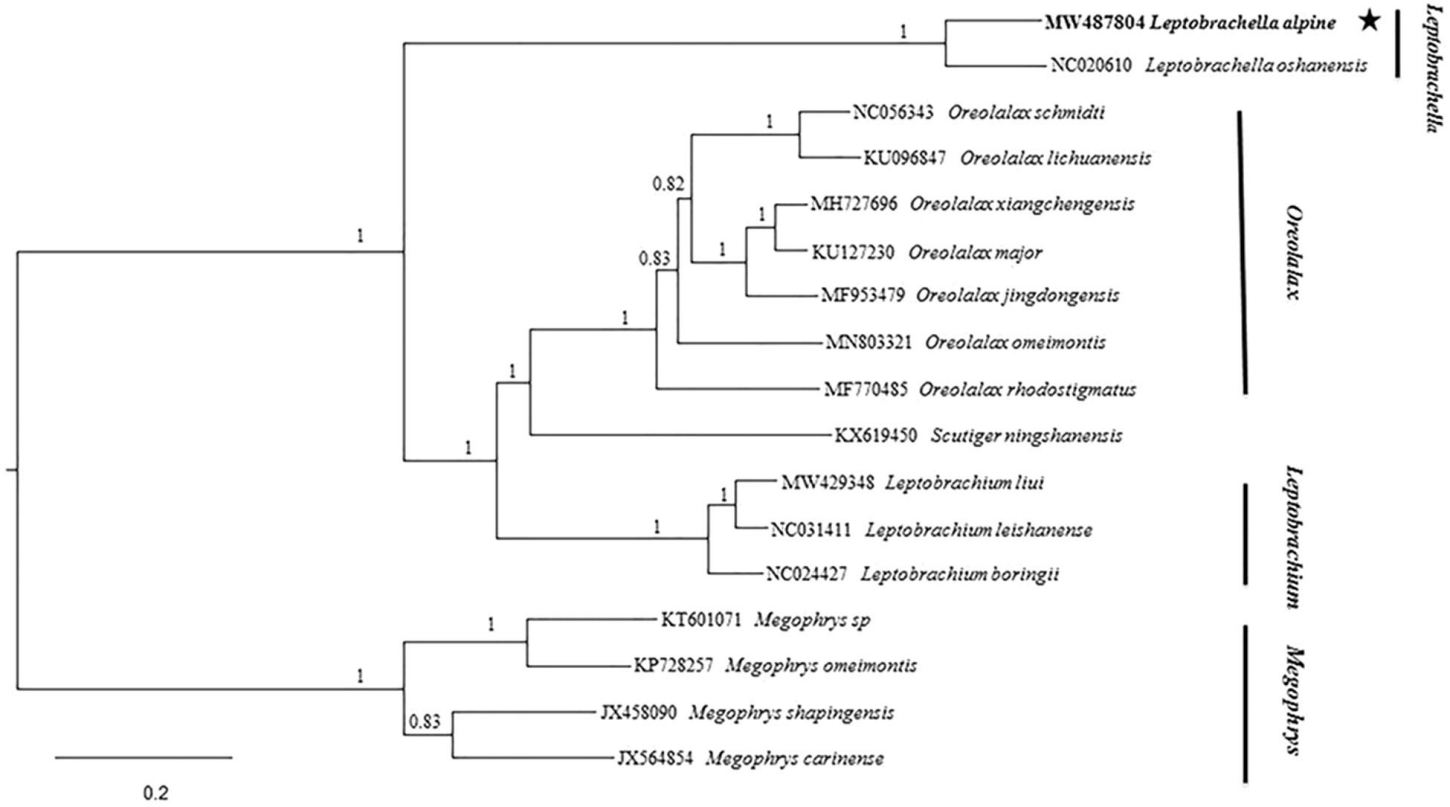
The Phylogenetic tree based on 17 mitogenome sequences of the family Megophryidae by Bayesian inference.

## Data Availability

The data that support the findings of this study are openly available in GenBank of NCBI at [https://www.ncbi.nlm.nih.gov] under the accession no. MW487804.

## References

[CIT0001] Fei L, Ye CY, Jiang JP. 2012. Colored Atlas of Chinese amphibians and their distributions. Chengdu (China): Sichuan Science and Technology Press.

[CIT0002] Kumar S, Stecher G, Tamura K. 2016. MEGA7: molecular evolutionary genetics analysis version 7.0 for bigger datasets. Mol Biol Evol. 33(7):1870–1874. 27004904 10.1093/molbev/msw054 PMC8210823

[CIT0003] Liang XX, Shu GC, Wang B, Jiang JP, Li C, Xie F. 2016a. Complete mitochondrial genome of the Leishan moustache toad, *Vibrissaphora leishanensis* (Anura: Megophryidae). Mitochondrial DNA B. 1(1):275–276. 10.1080/23802359.2016.1159937 PMC7871820 33644358

[CIT0004] Liang XX, Wang B, Li C, Xiang TM, Jiang JP, Xie F. 2016b. The complete mitochondrial genome of *Oreolalax major* (Anura: Megophryidae). Mitochondrial DNA B. 1(1):118–119. 10.1080/23802359.2016.1143339 PMC7871869 33644329

[CIT0005] Liu JB, Xue R, Wang Y, Li DY, Yan QG, Yang JD. 2015. The near-complete mitogenome sequence of the Omei horned toad *Megophrys omeimontis* Liu, 1950 (Anura, Megophryidae). Mitochondrial DNA. 27(4):2389–2390. 25856516 10.3109/19401736.2015.1028044

[CIT0006] Ronquist F, Teslenko M, van der Mark P, Ayres DL, Darling A, Höhna S, Larget B, Liu L, Suchard MA, Huelsenbeck JP. 2012. MrBayes 3.2: efficient Bayesian phylogenetic inference and model choice across a large model space. Syst Biol. 61(3):539–542. 22357727 10.1093/sysbio/sys029 PMC3329765

[CIT0007] Xiang TM, Wang B, Jiang JP, Li C, Xie F. 2013a. The complete mitochondrial genome of *Megophrys shapingensis* (Amphibia, Anura, Megophryidae). Mitochondrial DNA. 24(1):43–45. 22954176 10.3109/19401736.2012.717936

[CIT0008] Xiang TM, Wang B, Liang XX, Jiang JP, Li C, Xie F. 2013b. Complete mitochondrial genome of *Paramegophrys oshanensis* (Amphibia, Anura, Megophryidae). Mitochondrial DNA. 24(5):472–474. 23391261 10.3109/19401736.2013.766183

[CIT0009] Xu Q, Liu S, Wan R, Yue B, Zhang X. 2016. The complete mitochondrial genome of the *Vibrissaphora boringii* (Anura: Megophryidae). Mitochondrial DNA A DNA. 27(1):758–759. 10.3109/19401736.2014.915527 24841320

